# A clinicopathological analysis of supratentorial ependymoma, *ZFTA* fusion-positive: utility of immunohistochemical detection of *CDKN2A* alterations and characteristics of the immune microenvironment

**DOI:** 10.1007/s10014-023-00464-7

**Published:** 2023-06-16

**Authors:** Naohito Hashimoto, Tomonari Suzuki, Keisuke Ishizawa, Sumihito Nobusawa, Hideaki Yokoo, Ryo Nishikawa, Masanori Yasuda, Atsushi Sasaki

**Affiliations:** 1 Department of Pathology, Saitama Medical University Hospital, 38 Morohongou, Moroyama, Saitama 350-0495 Japan; 2 Department of Neuro-Oncology/Neurosurgery, Saitama Medical University International Medical Center, Hidaka, Saitama Japan; 3 Department of Human Pathology, Gunma University Graduate School of Medicine, Maebashi, Gunma Japan; 4 Department of Pathology, Saitama Medical University International Medical Center, Hidaka, Saitama Japan

**Keywords:** *CDKN2A* homozygous deletion, Tumor-associated macrophages, MTAP-p16-supratentorial ependymoma, *ZFTA* fusion-positive

## Abstract

EPN-ZFTA is a rare brain tumor where prognostic factors remain unclear and no effective immunotherapy or chemotherapy is currently available. Therefore, this study investigated its clinicopathological features, evaluated the utility of MTAP and p16 IHC as surrogate markers of *CDKN2A* alterations, and characterized the immune microenvironment of EPN-ZFTA. Thirty surgically removed brain tumors, including 10 EPN-ZFTA, were subjected to IHC. MLPA was performed for *CDKN2A* HD in 20 ependymal tumors, including EPN-ZFTA. The 5-years OS and PFS of EPN-ZFTA were 90% and 60%, respectively. *CDKN2A* HD was detected in two cases of EPN-ZFTA; these cases were immunohistochemically negative for both MTAP and p16 and recurred earlier after surgery. As for the immune microenvironment of EPN-ZFTA, B7-H3, but not PD-L1, was positive in all cases of EPN-ZFTA; Iba-1-positive or CD204-positive macrophages were large, while infiltrating lymphocytes were small, in number in EPN-ZFTA. Collectively, these results indicate the potential of MTAP and p16 IHC as useful surrogate markers of *CDKN2A* HD in EPN-ZFTA, and tumor-associated macrophages, including the M2 type, may contribute to its immune microenvironment. Furthermore, the expression of B7-H3 in EPN-ZFTA may indicate the usefulness of B7-H3 as a target of immune checkpoint chemotherapy for EPN-ZFTA via B7-H3 pathway.

**Supplementary Information:**

The online version contains supplementary material available at 10.1007/s10014-023-00464-7.

## Introduction

Ependymoma, *RELA* fusion-positive (EPN-RELA) was newly proposed in the WHO Classification of Tumours of the Central Nervous System (CNS), 2016 updated fourth edition (CNS4) [1] as an ependymoma (EPN) with a *C11orf95*-RELA fusion gene typically occurring in the supratentorial compartment. This tumor was then re-classified as supratentorial ependymoma, *ZFTA* fusion-positive (EPN-ZFTA) in the WHO Classification of Tumours of the CNS, 2021 fifth edition (CNS5) [2]. This tumor is relatively rare; a previous study reported only four cases (3.3%) of EPN-ZFTA among 122 ependymal tumors [3]. EPN-ZFTA frequently occurs in children with male predominancy [4]. Diagnostic criteria include a supratentorial occurrence as well as morphological and immunohistochemical features of EPN, and the detection of a fusion gene involving *ZFTA* by reverse transcriptase polymerase chain reaction (RT-PCR) or fluorescence in situ hybridization (FISH) is essential for the diagnosis of EPN-ZFTA [2]. Recent studies reported that immunohistochemistry (IHC) for L1 cell adhesion molecule (L1CAM) or nuclear factor kappa-light-chain-enhancer of activated B cells (NFκB) is also useful for the diagnosis of EPN-ZFTA [5–7]. Surgical resection and adjuvant radiotherapy are standard treatments, while there is currently no effective chemotherapy. The extent of resection was previously reported to be a prognostic factor, and a Ki-67 (MIB-1) labeling index (LI) < 10% was also associated with a better prognosis [6, 8]; however, there are no established prognostic factors available for EPN-ZFTA.

A *cyclin-dependent kinase inhibitor 2A* (*CDKN2A*) homozygous deletion (HD) has been detected in EPN-ZFTA and the prognosis of EPN-ZFTA harboring *CDKN2A* HD was shown to be poorer [9–11]. Although multiplex ligation-dependent probe amplification (MLPA) is useful for detecting *CDKN2A* HD, this procedure is too complex [12] to be universally available. The utility of methylthioadenosine phosphorylase (MTAP) IHC as a surrogate marker of *CDKN2A* HD in astrocytic tumors has been demonstrated [12], as has been that of p16 IHC in a few EPN-ZFTA cases [11]. To the best of our knowledge, MTAP and p16 IHC as surrogate markers of EPN-ZFTA has not yet been analyzed.

A previous study on EPN reported relationships between the immune microenvironment and prognosis as well as between prognosis and lymphocyte/macrophage infiltration [13]. However, the expression of programmed cell death-ligand 1 (PD-L1) was not identified as a prognostic factor [13]. On the other hand, the expression of B7 homolog 3 protein (B7-H3, CD276) was identified as an immune checkpoint molecule for glioblastoma (GBM), but not for EPN-ZFTA [14]. Tumor-associated macrophage (TAM) predicts poor prognosis in high-grade glioma [15]; in a similar vein, the prognostic role of TAM was also reported for EPN [13]. However, only a few studies are available about the immune microenvironment of EPN-ZFTA [13], and quantitative analyses of TAM among ependymal tumor subtypes, which would be of great help for understanding the immune microenvironment of ependymal tumors, have not been reported.

Therefore, the aims of the present study were as follows: (1) to clarify the clinicopathological characteristics of EPN-ZFTA, including overall survival (OS) and progression-free survival (PFS), in a single institution in Japan, (2) to evaluate the utility of MTAP and p16 IHC as surrogate markers of *CDKN2A* HD in EPN-ZFTA, and (3) to immunohistochemically characterize immune cells and immunological molecules in the microenvironment of EPN-ZFTA.

## Materials and methods

### Patients and tumor materials

Thirty tumors surgically removed at the Saitama Medical University International Medical Center and Saitama Medical University Hospital, Japan, between April 2008 and March 2020 were analyzed in the present study. Samples included 10 EPN-ZFTA, 4 spinal EPN, NOS (SC-EPN), 6 posterior fossa group A EPN (PFA-EPN), and 10 GBM (7 GBM, IDH-wildtype; 2 GBM, NOS; and 1 IDH-mutant astrocytoma, CNS WHO grade 4) according to CNS5. *ZFTA* fusion genes were detected in all EPN-ZFTA cases by FISH and/or RT-PCR. We graded the histological degree of malignancy of EPN-ZFTA by the presence of both microvascular proliferation (MVP) and brisk miotic activity in accordance with CNS5. The present study was approved by the Institutional Review Board of Saitama Medical University Hospital (No. 20030.01).

### IHC

IHC was performed on 3–4-μm-thick sections from formalin-fixed, paraffin-embedded (FFPE) tissue blocks as follows: the primary antibodies used included those for CD3 (PS1, 1: 100, Novocastra, Newcastle Upon Tyne, UK), CD4 (1F6, 1: 40, Novocastra, Newcastle Upon Tyne, UK), CD8 (C8/144B, 1: 50, Dako, Santa Clara, CA, USA), CD20 (L26, 1: 100, Dako, Santa Clara, CA, USA), MTAP (2G4, 1: 200, Abnova, Taipei, Taiwan), p16 (G175-405, 1: 10, BD Biosciences, Franklin Lakes, NJ, USA), CD204 (SRA-E5, 1: 25, Trans Genic Inc., Fukuoka, Japan), Iba-1 (013-27691, 1: 1000, Fujifilm Wako, Osaka, Japan), NFκB p65 (D14E12, 1:400, Cell Signaling Technology, Inc., Danvers, MA, USA), and B7-H3 (AF1027, 1: 200, R&D systems, Minneapolis, MN, USA). In the present study, two types of PD-L1 antibodies were used: PD-L1 (28-8, 1:200, Abcam, Cambridge, UK) and PD-L1 (E1L3N, 1:300, Cell Signaling Technology, Inc., Danvers, MA, USA). An antigen retrieval pretreatment included autoclaving (121 °C for 10 min) in Histofine antigen solution (pH9, 95 °C for 40 min; Nichirei. Tokyo, Japan) for CD3, CD4, CD8, CD20, MTAP, and p16 as well as in citrate buffer solution (pH6.0) for NFκB p65, CD204, and Iba-1. Regarding the two PD-L1 antibodies, autoclaving (95 °C for 30 min) in citrate buffer solution (pH6.0) and EDTA (pH9) was used for the clones 28-8 and E1L3N, respectively. Immunoreactivity was visualized with Rabbit Rinker (Dako) for the PD-L1 antibodies, while the two-step polymer detection system (MULTI) (Nichirei, Tokyo, Japan) was used for the other antibodies. Immunostained sections were visualized with diaminobenzidine as the chromogen.

### MLPA

MLPA was performed to detect *CDKN2A* HD in all cases of EPN-ZFTA, SC-EPN, and PFA-EPN. DNA samples were collected from FFPE samples. The P370 MLPA kit (MRC Holland, Amsterdam, the Netherlands) was selected as the best test kit for the detection of *CDKN2A* HD. MLPA was performed as previously reported [16, 17]. We identified *CDKN2A* HD in a tumor when the probe ratio was less than 0.5, and the *CDKN2A* hemizygous deletion when it was between 0.5 and 0.8. A probe ratio ≥ 0.8 was defined as normal [18].

### Quantitative analysis of IHC

We performed MTAP and p16 IHC on the ten cases of EPN-ZFTA. A quantitative analysis of immunohistochemical products was conducted by the three authors (NH, KI, AS), including two neuropathologists (KI, AS), with all three being blinded to the *CDKN2A* genetic abnormality. The expression of p16 was considered to be negative when the staining of the nucleus was completely absent and positive when it was detected in at least 1% of tumor cell nuclei [9]. MTAP is normally expressed in the cytoplasm of normal cells, such as epithelial cells and lymphocytes. We considered MTAP immunostaining to be positive when the tumor cell cytoplasm was clearly positive relative to physiologically positive cells and negative when the tumor cell cytoplasm was clearly not positive relative to normal expression in the cytoplasm of positive cells. Cases in which the expression of MTAP was absent in physiologically positive cells [12] were excluded from the quantitative analysis.

A quantitative analysis of Iba-1 and CD204 was performed on ten cases of EPN-ZFTA, 4 of SC-EPN, 6 of PFA-EPN, and 10 of GBM. Sections were observed under a magnification of × 200, and five microscopic fields with the largest positive area were evaluated. Necrosis was excluded from the analysis because the immunoproduct commonly observed there was considered to be non-specific and, thus, a false positive.

A correlation analysis between CD8 and B7-H3 was performed on ten cases of EPN-ZFTA. In subsequent quantitative analyses, digital photos were taken using an all-in-one fluorescence microscope (BZ-X810, KEYENCE, Osaka, Japan).

We identified an area containing the highest population of CD8-positive lymphocytes at a magnification of × 200, captured five microscopic fields, and counted positive cells manually in this area. B7-H3 expression was also assessed in the same area: its expression was observed at a magnification of × 200, five microscopic fields were captured, and the positive area per microscopic field was calculated using BZ-X Analyzer software (BZ-H4A, KEYENCE, Osaka, Japan). The relationship between CD8 and B7-H3 was then analyzed.

### Statistical analysis

We used JMP ver.16 (SAS Institute, Cary, NC, USA) for statistical analyses. Kaplan–Meier curves were drawn for OS and PFS in patients with EPN-ZFTA. Differences in the expression of Iba-1 and CD204 among EPN-ZFTA, SC-EPN, PFA-EPN, and GBM were assessed and schematically visualized using a boxplot. We used the Steel–Dwass test to assess the significance of differences. The relationship between B7-H3 and CD8 was examined using bivariate analyses. A P-value < 0.05 was considered to be significant.

## Results

### Clinical and pathological features

#### Clinical features of EPN-ZFTA

The clinical characteristics of EPN-ZFTA are summarized in Table 1. The frequency of EPN-ZFTA in ST-EPN studied was 33% (10/30). The 10 EPN-ZFTA cases were predominantly male (male: 7, female: 3). Age at onset ranged between 1 and 63 years (median: 6 years, mean: 12.7 years). Tumor locations varied within the supratentorial compartment as follows: frontal in two cases, temporal in 1, parietal in 4, and occipital in 3. Cysts were detected in eight out of nine cases. Gross total resection (GTR) or subtotal resection (STR) was performed on all cases, followed by adjuvant radiotherapy. Recurrence was observed in seven cases, and two died of disease progression. Lung metastasis was detected in one autopsied case (case 1).

**Table 1:** Clinical characteristics of superatentorial ependymoma, *ZFTA* fusion-positive

No	Age at onset (year)	Sex	Location	Imaging	Treatment	Month to recurrence	Outcome	Follow-up (months)
1	21	Male	lt-parietal, lung metastasis	Unavailable	STR + RT( 54 Gy) + CT* STR + CT**	22	Dead	56
2	11	Male	rt-frontal	Cyst( +)	GTR + RT( 59.4 Gy)* RT( 27 Gy)**	39	Alive	39
3	5	Male	lt-parietal	Cyst( +)	GTR + RT( 59.4 Gy)* GTR + RT( 54 Gy)**	83	Dead	89
4	63	Female	lt-frontal	Cyst( +)	STR + RT( 60 Gy)* CT**	25	Alive	121
5	7	Male	lt-occipital	Cyst(-)	GTR + RT( 59.4 Gy)*	-	Alive	37
6	5	Male	rt-occipital	Cyst( +)	GTR* GTR + RT( 59.4 Gy)**	51	Alive	85
7	1	Female	lt-parietal	Cyst( +)	STR + RT( 54 Gy) + GTR*	-	Alive	131
8	8	Female	rt-temporal	Cyst( +)	STR + RT( 59.4 Gy)* GTR**	107	Alive	110
9	3	Male	rt-occipital	Cyst( +)	GTR + RT( 59.4 Gy) + CT*	-	Alive	99
10	3	Male	lt-parietal	Cyst( +)	STR + RT (50 Gy)* GTR + CyberKnife( 30 Gy) + CT**	110	Alive	143

*lt* left, *rt* right, *STR* subtotal resection, *GTR* gross total resection, *RT* radiation therapy, *CT* chemotherapy

^*^Treatment at onset

^**^Treatment at recurrene

Kaplan–Meier curves for OS and PFS in patients with EPN-ZFTA are shown in Supplemental Fig. 1. Five-year OS and PFS rates were 90% (9/10 cases) and 60% (6/10 cases), respectively. Median PFS was 82 months. Among surviving cases, three were free of disease after the initial treatment for 37, 99, and 131 months, respectively. In the two cases that died, the intervals from the onset to death were 89 and 56 months, respectively. The median OS in GBM and PFA-EPN was 15 and 21 months, respectively. The median PFS in GBM and PFA-EPN was 11 and 9 months, respectively. No deaths or recurrences were observed during the follow-up period for SC-EPN.

**Fig. 1:**
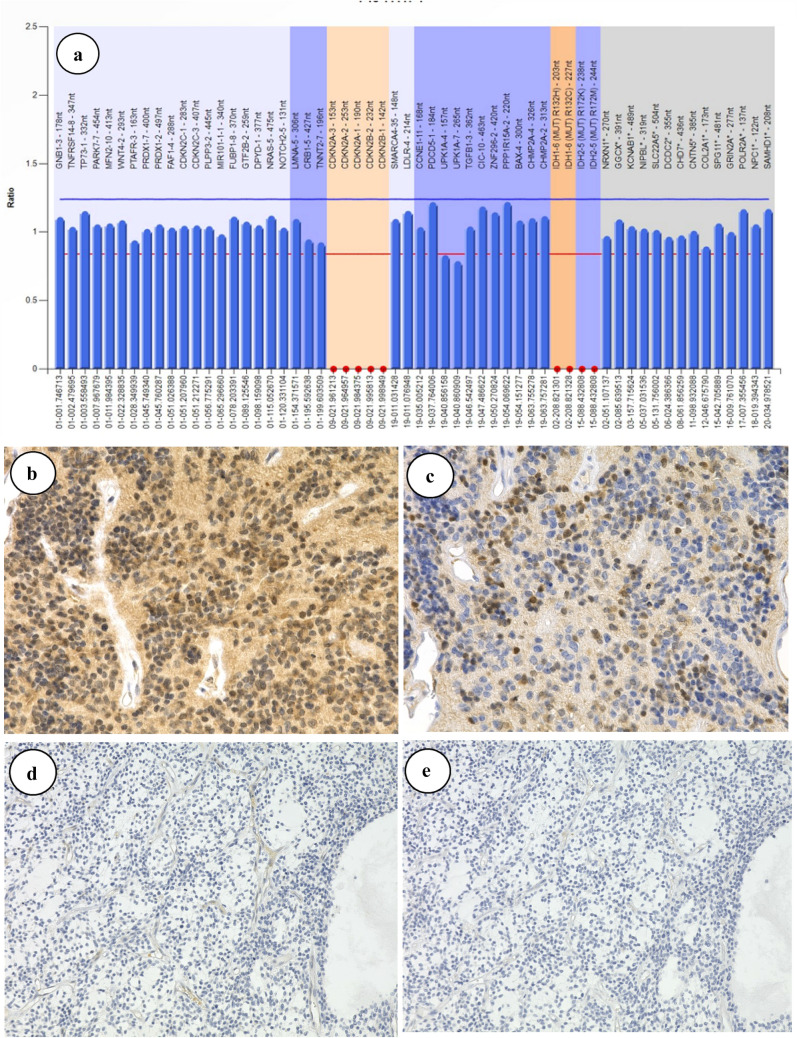
The *CDKN2A* copy number status and immunohistochemistry for MTAP and p16 in representative cases of EPN-ZFTA. Case 2 shows the *CDKN2A* homozygous deletion (HD) by MLPA (**a**). A case of EPN-ZFTA with normal *CDKN2A* shows retained MTAP cytoplasmic (**b**, × 400) or p16 nuclear (**c**, 400 ×) staining. A case of EPN-ZFTA with *CDKN2A* HD shows the loss of MTAP cytoplasmic (**d**, 400 ×) and p16 nuclear (**e**, 400 ×) staining (case 2)

#### Pathological features of EPN-ZFTA

The pathological characteristics of EPN-ZFTA are summarized in Table 2. Regarding the primary tumor, 3 cases were classified as Grade 2, and 6 as Grade 3. MIB-1 LI for EPN-ZFTA was as follows (range, median, mean): 5.9–62.2%, 18.8%, 24.6% for primary, and 10.3–55.0%, 16.6%, 24.7% for recurrent. MIB-1 LI was higher in the recurrent group than in the primary group. Histological features, such as MVP (primary: 6/9, recurrent: 2/4), necrosis (primary: 6/9, recurrent: 3/4), and clear cells (primary: 6/9, recurrent: 3/4), were observed in EPN-ZFTA. One case of EPN-ZFTA (case 2) exhibited a polymorphous histology, such as epithelioid, spindle, and small tumor cells (Supplemental Fig. 2). One case with *CDKN2A* HD was classified as Grade 3, with MVP but without necrosis or clear cells (case 1). The other case with *CDKN2A* HD was classified as Grade 2, without MVP or necrosis but with clear cells (case 2).

**Table 2:** Pathological features of supratentorial ependymoma, *ZFTA* fusion-positive

No	Age	Grade	MVP	Necrosis	Clear cells	MIB-1 LI (%)
1	21*	3*	+ *	-*	-*	24.2*
2	11*	2*	-*	-*	+ *	62.2*
3	5* 12**	2* 3**	-* + **	+ * + **	+ * + **	8.6* 55.0**
4	63*	3*	+ *	+ *	+ *	15.8*
5	7*	3*	+ *	+ *	-*	18.8*
6	5* 10**	2* 2**	-* -**	-* -**	+ * + **	5.9* 10.5**
7	1*	3*	+ *	+ *	+ *	26.3*
8	8* 16**	3* 3**	+ * + **	+ * + **	+ * + **	6.6* 10.3**
9	3*	3*	+ *	+ *	-*	53.1*
10	12**	2**	-**	+ **	-**	22.8**

Grades were defined according to the WHO 2021. The recurrent tumors of cases 1, 2, 4 and the primary tumor of case 10 were not available for this array of pathological analysis

*MVP* microvascular proliferation, *MIB-1 LI* MIB-1 labeling index

^*^Primary tumor

^**^Recurrent tumor

+, positive; -, negative

### The *CDKN2A* copy number status and MTAP and p16 IHC

MLPA revealed *CDKN2A* HD in two cases (Fig. 1a). One case presented with lung metastasis and died of the disease (case 1), while the other relapsed 39 months after being diagnosed (case 2). The hemizygous deletion of *CDKN2A* was observed in three cases (cases 3–5), including one that died of the disease. *CDKN2A* remained intact in five cases (cases 6–10).

The expression of MTAP was observed in six cases (Fig. 1b) and that of p16 in 7 (Fig. 1c). The loss of MTAP expression was noted in 40% (4/10) of EPN-ZFTA cases (Fig. 1d). The loss of the nuclear expression of p16 was detected in 30% (3/10) of EPN-ZFTA cases (Fig. 1e). Two cases with *CDKN2A* HD showed the loss of MTAP and p16.

Comparisons of immunohistochemical staining for MTAP and p16 with the *CDKN2A* genetic abnormality showed that the two cases with *CDKN2A* HD were negative for both MTAP and p16 (Table 3). Two out of the three cases with the *CDKN2A* hemizygous deletion (cases 3, 5) expressed MTAP or p16. The other case showed uneven nuclear expression for p16 (case 4). Four out of the five cases in which *CDKN2A* remained normal (cases 7–10) expressed both MTAP and p16. The expression of p16 in cases with an abnormal *CDKN2A* gene was positive even at a low magnification.

**Table 3:** MLPA of CDKN2A and immunohistochemical staining for MTAP and p16 in supratentorial ependymoma, *ZFTA* fusion-positive cases

No	Age at onset (year)	Sex	*CDKN2A *(MLPA)	p16	MTAP
1	21	Male	Homo del	-	-
2	11	Female	Homo del	-	-
3	5	Male	Hemi del	+	-
4	63	Female	Hemi del	±	+
5	7	Male	Hemi del	-	+
6	5	Male	Normal	+	-
7	1	Female	Normal	+	+
8	8	Female	Normal	+	+
9	3	Male	Normal	+	+
10	3	Male	Normal	+	+

Homozygous deletion, probe ratio < 0.5

Hemizygous deletion, probe ratio 0.5 to 0.8

Normal, probe ratio ≥ 0.8

-, loss of expression

+, uniform nuclear expression for p16, cytoplasmic expression for MTAP

± , uneven nuclear expression

We also performed MLPA on four cases of SC-EPN and six of PFA-EPN; *CDKN2A* HD was absent in these cases (data not shown). Among these cases, the expression of MTAP was observed in eight and that of p16 in four. One case showed inadequate staining for MTAP.

### Immune microenvironment

#### Macrophage markers

**Fig. 2:**
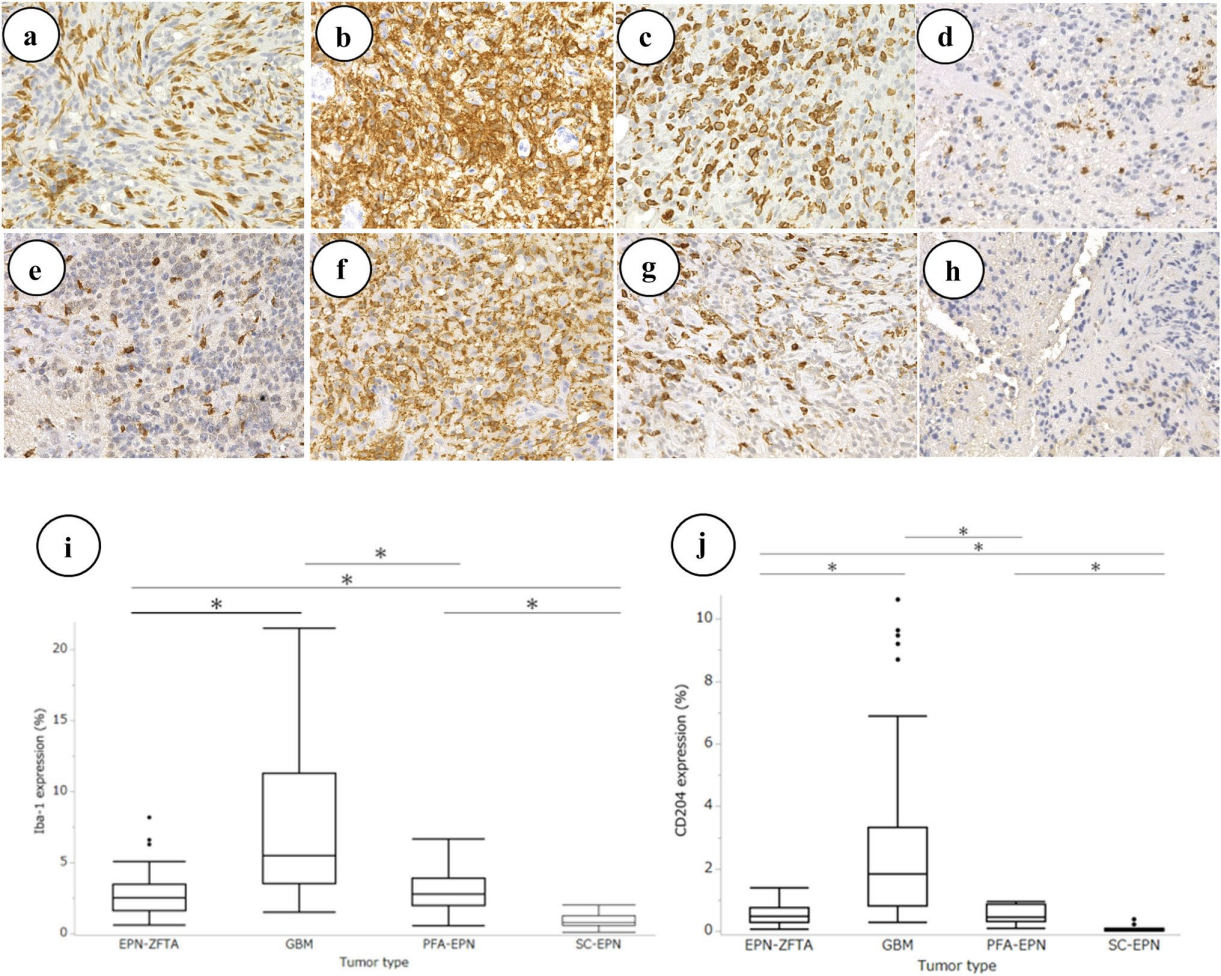
The expression of macrophage markers in cases of EPN-ZFTA, GBM, PFA-EPN, and SC-EPN. **a**–**h** Iba-1 expression is observed in cases of EPN-ZFTA (**a**, × 400), GBM (**b**, × 400), PFA-EPN (**c**, × 400), and SC-EPN (**d**, × 400). CD204 expression is observed in cases of EPN-ZFTA (**e**, × 400), GBM (**f**, × 400), PFA-EPN (**g**, × 400), and SC-EPN (**h**, × 400). **i**, **j** The X-axis indicates tumor types. The Y-axis indicates the range of expression for Iba-1 and CD204 per high power field. The median expression of Iba-1 is (**i**) 2.54% (EPN-ZFTA), 5.52% (GBM), 2.8% (PFA-EPN), and 0.81% (SC-EPN); the median expression of CD204 is (**j**) 0.47% (EPN-ZFTA), 1.83% (GBM), 0.46% (PFA-EPN), and 0.025% (SC-EPN); combinations where significant differences were noted are also indicated (*P < 0.05)

The expression of Iba-1 and CD204 varied in all cases of EPN-ZFTA, GBM, PFA-EPN, and SC-EPN (Fig. 2a–h). We compared positivity for Iba-1 and CD204 in cases of EPN-ZFTA, GBM, PFA-EPN, and SC-EPN using a computer-assisted image analysis. The positive ratio varied by tumor types with the highest expression of Iba-1 and CD204 being observed in GBM. The expression of Iba-1 and CD204 was similar between EPN-ZFTA and PFA-EPN. The lowest expression of Iba-1 and CD204 was noted in SC-EPN. Significant differences in the expression of Iba-1 and CD204 were detected in the following combinations: GBM and EPN-ZFTA, GBM and PFA-EPN, EPN-ZFTA and SC-EPN, and PFA-EPN and SC-EPN (all P < 0.05). The results obtained for each tumor type are summarized using box-plots (Fig. 2i, j). The expression of Iba-1 and CD204 was slightly elevated when MVP was present.

#### Lymphocyte markers and immune checkpoint inhibitors

CD3-positive lymphocytes were sporadically noted, and CD8-positive lymphocytes were equally noted to CD3-positive lymphocytes, in nine cases of EPN-ZFTA, including the two cases with *CDKN2A* HD. CD4-positive lymphocytes were negative in all cases. CD20-positive lymphocytes were present in three cases, including the one with *CDKN2A* HD (case 1); however, the number of positive cells was small even in the field with the highest population (Supplemental Fig. 3a–d).

PD-L1 expression was analyzed using two IHC clones, 28-8 and E1L3N. The expression of PD-L1 was not observed in any of the EPN-ZFTA, SC-EPN, PFA-EPN, and GBM cases, which was in sharp contrast to its expression in the positive controls of tonsil tissue and lung adenocarcinoma (Supplemental Fig. 3e–g).

The diffuse and strong expression of B7-H3 was observed in 8 out of 10 GBM cases. B7-H3 was also expressed in all cases of EPN-ZFTA (Supplemental Fig. 3h). B7-H3 was positive in MVP, but was negative in non-MVP vessels. B7-H3 was expressed in a small number of neoplastic cells in 3 cases of EPN-ZFTA.

**Fig. 3:**
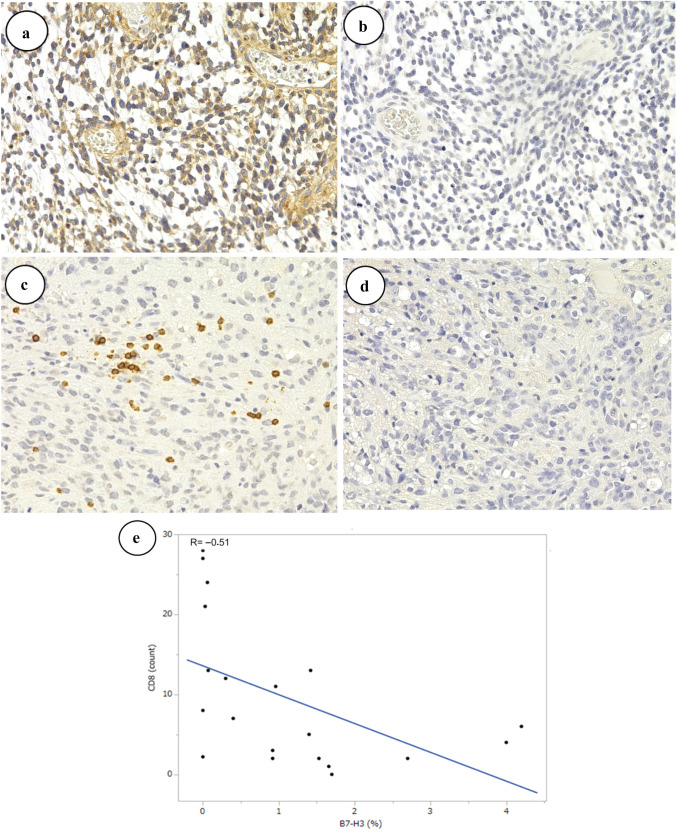
Relationships between B7-H3 and CD8 in EPN-ZFTA. **a**–**d** In the presence of high B7-H3 expression levels (**a**, × 400, case 5), the expression of CD8 decreased or was lost (**b**, × 400, case 5). In the presence of high CD8 expression levels (**c**, × 400, case 5), the expression of B7-H3 decreased or was lost (**d**, × 400, case 5). **e** The X-axis indicates the ratio of the B7-H3-positive area/microscopic field area. The Y-axis indicates the number of CD8-positive cells/microscopic field. A negative correlation was observed between B7-H3 and CD8 (R = − 0.51, P = 0.0204)

We examined the relationship between the expression of CD8 and B7-H3 in EPN-ZFTA. High expression levels of B7-H3 were associated with low expression levels of CD8 (Fig. 3a, b), and vice versa (Fig. 3c, d). A negative correlation was observed between CD8 and B7-H3 (Fig. 3e) (R = − 0.51, P = 0.0204).

## Discussion

The clinicopathological features of EPN-ZFTA in previous studies were compared with those in the present study. In our study, cysts were observed in 8/9 cases, which was higher than that (12/19 cases) in a previous study on EPN-ZFTA [4]. Although male or pediatric predominancy, which was similar to previous studies, was also observed, it is important to note that this predominancy remains controversial [19, 20]. The frequency of EPN-ZFTA among ST-EPN was 33% (10/30) in the present study, which was similar to a previous one reporting 40% (17/42) [20].

The prognosis of EPN-ZFTA has not yet been established. According to Branger et al. [4], 5-years PFS and OS rates were 67.5 and 72.2%, respectively. Pajtler et al. [21] reported 5-years PFS and OS rates of 29 and 75%, respectively, and concluded that patients with EPN-RELA (EPN-ZFTA) had a dismal prognosis. Although the prognosis of EPN-ZFTA was better in the present study, the number of cases examined was small. Although the treatment, such as GTR/STR and radiotherapy, may underlie the favorable prognosis noted in the present study, it is difficult to explain why the prognosis of patients differed between the present study and previous ones because the details of treatment thereof were not described. In addition, previous studies did not search for *CDKN2A* gene abnormalities, which may have been associated with the worse prognosis of patients. Although a relationship was previously reported between Ki-67 LI ≥ 7% and the poor prognosis of patients with EPN [22], other studies suggested that the WHO grading and MIB-1 LI were not associated with a poor prognosis [8, 21]. Therefore, the prognostic value of proliferative indices, such as MIB-1 LI and the WHO grading, remains still unestablished. In the present study, all cases classified as WHO grade 2 relapsed, whereas 50% (3/6 cases) classified as WHO grade 3 did not. Moreover, all cases with low MIB-1 LI (< 8.6%) relapsed, whereas 42% (3/7 cases) with high MIB-1 LI (≥ 18.8%) did not. Therefore, the present results highlight the difficulty of using MIB-1 LI and/or the WHO grading as prognostic factors for future recurrence. The median OS of GBM and PFA-EPN was worse than that of EPN-ZFTA, and no deaths or recurrences were observed for SC-EPN in the present study. In general, it was reported that a better prognosis was observed for SC-EPN compared to EPN-ZFTA and PFA-EPN [23]; however, it should be noted that the number of cases is too small in the present study, especially for SC-EPN and PFA-EPN. The prognosis of GBM is, as is sufficiently expected, miserable compared to SC-EPN, PFA-EPN and EPN-ZFTA [24].

The present study indicates the utility of MTAP and p16 IHC as surrogate markers for *CDKN2A* HD [12]. MTAP is the key enzyme in the methionine salvage pathway [10] and is normally expressed in various tissues; however, its expression was previously shown to be lost in some tumors [25, 26]. The p16 protein plays an important role in blocking the G1 to S phase transition via the inhibition of CDK4 and CDK6 [12, 27] and is encoded by *CDKN2A* (9p21). The gene coding MTAP is located on 9p21, which is only 165 kb telomeric to *CDKN2A*. In a previous study, IHC revealed that GBM cases with *CDKN2A* HD did not express p16 [9]. Satomi et al. [12] showed the potential of the deficiency of MTAP expression by IHC as a predictive surrogate marker for *CDKN2A* HD in IDH-mutant astrocytomas. In the present study, we examined MTAP and p16 IHC in EPN-ZFTA with or without *CDKN2A* HD, and found that neither MTAP nor p16 was expressed in the two cases with *CDKN2A* HD. The three cases that were positive for MTAP or p16 harbored the *CDKN2A* hemizygous deletion. The relationship between the *CDKN2A* hemizygous deletion and the expression of MTAP or p16 has not yet been reported for ependymal tumors. The frequency of *CDKN2A* HD in ST-EPN was previously reported to be 9.6% (3/31 cases) [28]. In the present study, the frequency of *CDKN2A* HD in EPN-ZFTA was higher at 20% (2/10 cases). Since non-*ZFTA* fusion cases were included in the previous study, the frequency of *CDKN2A* HD may have been lowered by the inclusion of non-*ZFTA* fusion cases. *CDKN2A* HD was previously, in fact, detected in EPN-ZFTA [10]; Junger et al. [10] detected *CDKN2A* HD in 9 out of 54 (16.7%) EPN-ZFTA cases, which is consistent with the present results. On the other hand, extracranial metastasis is rare in ependymal tumors. In a previous study that searched 258 cases of EPN, extracranial metastasis was only observed in 5 cases, which was equivalent to 2% [29]. *CDKN2A* HD was also detected in a case of extracranial metastasis including lung metastasis [30]. In the present study, one case (case 1) had lung metastasis and also harbored *CDKN2A* HD. The other case with *CDKN2A* HD (case 2) developed recurrence at a relatively early stage after surgery, despite the fact that there was no MVP or necrosis, being histologically classified into WHO grade 2. In conjunction with an animal study on adult mice showing a significantly worse prognosis in those with *CDKN2A* HD [31], these results indicate a relationship between *CDKN2A* HD and the poor prognosis of EPN-ZFTA. Regardless of the morphological features such as MVP, necrosis, and the WHO grading, EPN-ZFTA with *CDKN2A* HD showed a poor prognosis represented by death or early recurrence in the present study; furthermore, characteristic histological features indicating the presence of *CDKN2A* HD were not identified in these EPN-ZFTA cases. These findings suggest the necessity of searching for *CDKN2A* HD regardless of the histological features or the WHO grading in EPN-ZFTA. Based on previous findings and the present results, we propose a practical flowchart for the stratification of EPN-ZFTA (Supplemental Fig. 4), which is applicable to the routine diagnostic practice of EPN-ZFTA. Cases expressing MTAP and p16 may be regarded as non-*CDKN2A* HD; MLPA to detect *CDKN2A* HD is thus unnecessary. Cases with the loss of both MTAP and p16 expression may be regarded as *CDKN2A* HD; although MLPA to detect *CDKN2A* HD is not necessary, it is better to perform it to confirm IHC results. On the other hand, MLPA is essential for the following cases: (1) negative for MTAP or p16, and (2) showing inadequate immunostaining for MTAP (for example, when physiologically positive cells are also negative) or p16 (when nuclear staining is uneven and, thus, assessments with a low magnification are difficult).

A previous study confirmed that PD-L1 IHC, such as SP263, was expressed on tumor cell membranes, but not on immune cells [32]. Among L1CAM-positive ST-EPN cases, 40% (6/15 cases) were positive for SP263 and 20% (3/15 cases) for E1L3N. The latter antibody, E1L3N, which was used in the present PD-L1 IHC study, yields positive immunoproducts on tumor cell membranes [13]. Another study clearly identified PD-L1 in supratentorial EPN-RELA using various techniques, including Western blotting, flow cytometry, and IHC, and all ten cases were positive for SP263 [33]. The reason for the absence of PD-L1 immunopositivity in the present study remains unclear; however, it is unlikely to be due to insufficient immunohistochemical techniques because we detected the expression of PD-L1 in positive control tissue sections.

B7-H3, also known as CD276, is a transmembrane protein from the B7 family that was initially reported in 2001 [34]. It is regarded as a third group of immune checkpoints [35]. Although the ligand for B7-H3 has not yet been identified, it is known to be expressed on T-cells, B-cells, and dendritic cells [34, 36]. The expression of B7-H3 was previously shown to be higher in the tumor vascular endothelium than in the normal endothelium and was associated with tumor proliferation [37, 38]. B7-H3 expression was detected in cases of medulloblastoma and GBM and correlated with a worse prognosis [14, 39, 40]. Although the expression of B7-H3 has not yet been reported in EPN-ZFTA, we herein confirmed its expression in EPN-ZFTA, indicating possible involvement of the immune checkpoint molecules in EPN-ZFTA, and found an inverse correlation between its expression levels and the number of infiltrating CD8-positive lymphocytes. CD8-positive lymphocytes are cytotoxic T-cells associated with a favorable prognosis in gliomas [13]. As CD8-positive lymphocytes were suppressed by immunosuppressive function of B7-H3 as was reported previously [41], the inverse correlation between CD8-positive lymphocytes and B7-H3 would indicate the usefulness of B7-H3 as a target of immune checkpoint chemotherapy for EPN-ZFTA via B7-H3 pathway. Similar findings have been reported for GBM [14]. In GBM, the expression of MYC regulates cell differentiation and high expression levels were associated with high tumorigenicity [40]. Another study confirmed that the knockdown of B7-H3 regulated the differentiation of GBM by modulating MYC expression [42]. Therefore, B7-H3, which modulates MYC expression, has potential as a target of immune checkpoint chemotherapy in EPN-ZFTA. Regarding lymphocyte markers such as CD3, CD8, and CD20 in the present study, the case of EPN-ZFTA without CD3-positive and CD8-positive lymphocytes (case 4) harbored relatively a favorable prognosis. On the other hand, two out of the three cases with CD20-positive lymphocytes also harbored relatively a favorable prognosis (cases 6, 8). While a probable better prognosis without CD3-positive and CD8-positive lymphocytes as in case 4 is inconsistent with a previous report [13], that with CD20-positive lymphocytes as in cases 6 and 8 is consistent with a previous report [13]. After all, the story behind B7-H3 and/or the presence or absence of lymphocytes in the immune microenvironment of EPN-ZFTA is likely not so straightforward, but given the present results, futher investigations into the prognostic values of B7-H3 and/or lymphocytes are warranted for EPN-ZFTA.

Macrophages infiltrating cancer tissues are called tumor-associated macrophages (TAMs). Iba-1 is a pan-histiocytic marker for both M1 and M2 macrophages, and CD204 is an M2 macrophage marker. While M1 TAMs are induced by lipopolysaccharide and interferon-γ stimulation, regulating acute inflammation, M2 TAMs exert anti-inflammatory effects, play roles in tissue remodeling and angiogenesis, and contribute to tumor growth [43–46]. Although TAMs were often historically described in the context of M1/M2 activation, single-cell RNA sequencing has revealed the complexity of macrophage responses, moving us beyond the linear M1/M2 activation paradigm in GBM [47]. Previous studies revealed that the infiltration of M2 macrophages was associated with the higher malignancy and worse prognosis of astrocytic tumors, including GBM [15, 48]. The number of TAMs in gliomas may be associated with the glioma type and grade; in a study that examined astrocytic tumors and oligodendrogliomas, the number of microglia was significantly higher in astrocytomas than in oligodendrogliomas, regardless of whether they were grade II or III [49]. Limited information is currently available on TAM in EPN. Nam, et al. [13] showed a high ratio of CD163/CD68 + cells (cut-off value of 1.007), indicating that M2 macrophages were associated with a poor prognosis in EPN patients. In previous studies, the prognosis in SC-EPN was better than PFA-EPN and EPN-ZFTA [22], and the prognosis of GBM was the worst among SC-EPN, PFA-EPN, and EPN-ZFTA [24], while the expression of Iba-1 and CD204 was the highest in GBM and the lowest in SC-EPN in the present study. The quantitative results of TAM among EPN tumor subtypes and GBM in the present study indicates the possibility for the prognostic value of TAM; this is because the possible correlation between poor prognostic tumor types and TAM expression was observed (Fig. 2). To confirm further the prognostic value of TAM in EPN-ZFTA, more detailed prognostic analyses in association with the amount of TAM expression would be an interesting issue. Moreover, a positive correlation between B7-H3 expression and M2 macrophage expression was reported in a previous study [50]. Therefore, a more detailed understanding of the relationship between B7-H3 expression and TAM subsets in the immune microenvironment of EPN-ZFTA will surely contribute to the development of new therapies for EPN-ZFTA**.**

As has been discussed above, the prognostic value of lymphocytes or TAMs is probable in EPN-ZFTA; in this regard, the association of lymphocytes or TAMs with *CDKN2A* HD, the latter of which is likely a potential prognostic determinant of EPN-ZFTA according to the present study, is an interesting issue. But the pathological behavior of T-lymphocytes/B-lymphocytes or TAMs was seemingly not different between EPN-ZFTA with *CDKN2A* HD and those without it. Furthermore, the small number of EPN-ZFTA with *CDKN2A* HD, which was just 2 in the present study, made it all the more difficult to evaluate the differences in the qualitative and quantitative behavior of T-lymphocytes/B-lymphocytes or TAMs among EPN-ZFTA cases with or without *CDKN2A* HD. Thus, the exact relationship between the immune microenvironment (lymphocytes or TAMs) in EPN-ZFTA and *CDKN2A* genetic abnormality still remains to be established. But since the issue of the immune microenvironment and *CDKN2A* abnormality in EPN-ZFTA, and its association with the prognosis of the tumor, are surely interesting, it awaits further studies in the future.

## Supplementary Information

Below is the link to the electronic supplementary material.

Supplementary file1 (DOCX 6944 KB)

## Data Availability

The datasets generated and/or analysed during the current study are available from the corresponding author on reasonable request.

## Abbreviations

B7-H3: B7 homolog 3 protein
CDKN2A/B: *Cyclin-dependent kinase inhibitor 2A/B*
CNS4: WHO Classification of Tumours of the Central Nervous System (CNS), 2016 updated fourth edition (CNS4)
CNS5: WHO Classification of Tumours of the CNS, 2021 fifth edition (CNS5)
EPN-RELA: Ependymoma, *RELA* fusion-positive
EPN-ZFTA: Supratentorial ependymoma, *ZFTA* fusion-positive
FFPE: Formalin-fixed, paraffin-embedded
FISH: Fluorescence in situ hybridization
GBM: Glioblastoma
GTR: Gross total resection
HD: Homozygous deletion
IHC: Immunohistochemistry
LI: Labeling index
L1CAM: L1 cell adhesion molecule
MLPA: Multiplex Ligation-dependent Probe Amplification
MTAP: Methylthioadenosine phosphorylase
MVP: Microvascular proliferation
NFκB: Nuclear factor kappa-light-chain-enhancer of activated B cells
PD-L1: Programmed cell death-ligand 1
PFA-EPN: Posterior fossa group A ependymoma
RT-PCR: Reverse Transcriptase Polymerase Chain Reaction
SC-EPN: Spinal ependymoma, NOS
ST-EPN: Supratentorial ependymoma
STR: Subtotal resection
TAM: Tumor-associated macrophage
