# Supplementary material for: A clinicopathological analysis of supratentorial ependymoma, ZFTA fusion-positive: utility of immunohistochemical detection of CDKN2A alterations and characteristics of the immune microenvironment
Source: Brain Tumor Pathol. 2023 Jun 16;40(3):163–75. doi: 10.1007/s10014-023-00464-7 (PMC10314846; doi:10.1007/s10014-023-00464-7)
Supplement: Supplementary file 1 — Supplementary file1 (DOCX 6944 KB) [file 10014_2023_464_MOESM1_ESM.docx]

Supplemental Fig 1.

Kaplan-Meier curves for OS (A) and PFS (B) in patients with EPN-ZFTA, GBM, PFA-EPN, and SC-EPN.

Supplemental Fig. 2.

EPN-ZFTA shows diverse histological features.

A case of EPN-ZFTA with *CDKN2A* HD is shown (case 2). Figures A to C are from the primary tumor and figures D to F from the recurrent tumor.

(A) Perivascular pseudorosette (×400).

(B) Epithelioid, clear cells (×400).

(C) Spindle cells. Note calcified deposits among them (×400).

(D) Small cells (×200).

(E) NFκBp65 is positive in tumor cell nuclei (NFκBp65 IHC) (×200).

(F) The MIB-1 labeling index is high (×200).

Supplemental Fig 3.

The expression of lymphocytic markers and immune checkpoint inhibitors in EPN-ZFTA.

CD3-positive (a, ×200 case 5), CD4-positive (b, ×200 case 5), and CD8-positive (c, ×200 case 5) lymphocytes are present, while CD20-positive (d, ×200 case 5) lymphocytes are absent. PD-L1 expression is observed in lung adenocarcinoma (e, ×200) as a positive control. PD-L1 expression is not present in EPN-ZFTA (f, ×200 case 5, clone 28-8; g, ×200 case 5, clone E1L3N). The diffuse expression of B7-H3 is noted in EPN-ZFTA (h, ×200 case 5). Figures (f, g, h) are from the same microscopic field.

Supplemental Fig 4.

A practical flowchart for the diagnosis of EPN-ZFTA using MTAP and p16 IHC as well as MLPA.

Cases with MTAP and p16 expression may be regarded as non-*CDKN2A* HD; MLPA to detect *CDKN2A* HD is not necessary. Cases with the loss of MTAP and p16 expression may be regarded as *CDKN2A* HD; MLPA to detect *CDKN2A* HD is not necessary, but it is better to perform it to confirm IHC results (dotted line). On the other hand, MLPA is essential for the following cases: 1) negative for MTAP or p16, and 2) inadequate immunostaining for MTAP (for example, when physiologically positive cells are also negative) or p16 (when nuclear staining is uneven and, thus, assessments with a low magnification are difficult).


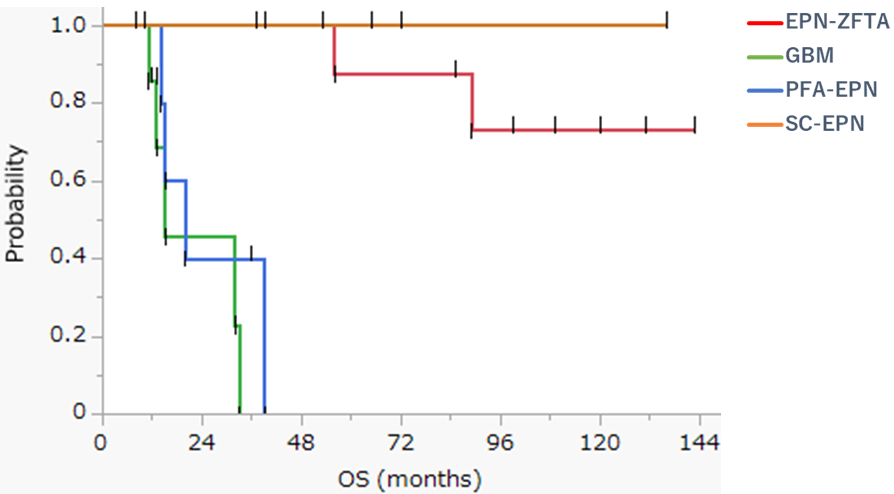

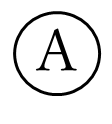

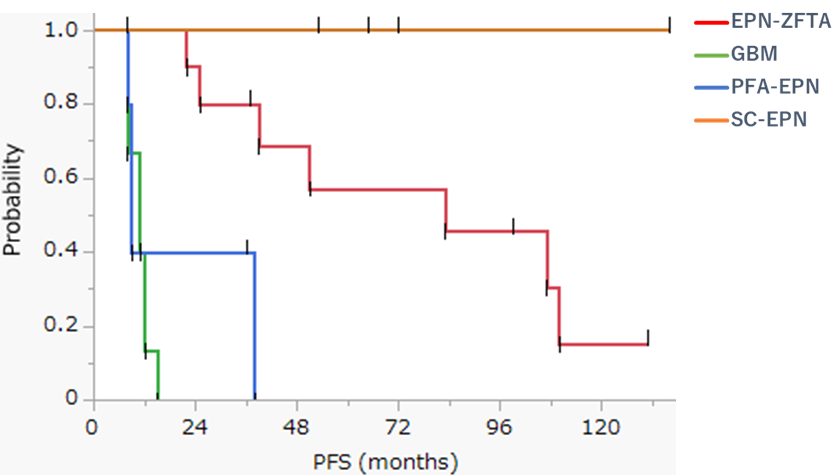

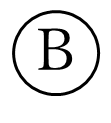


Supplemental Fig. 1


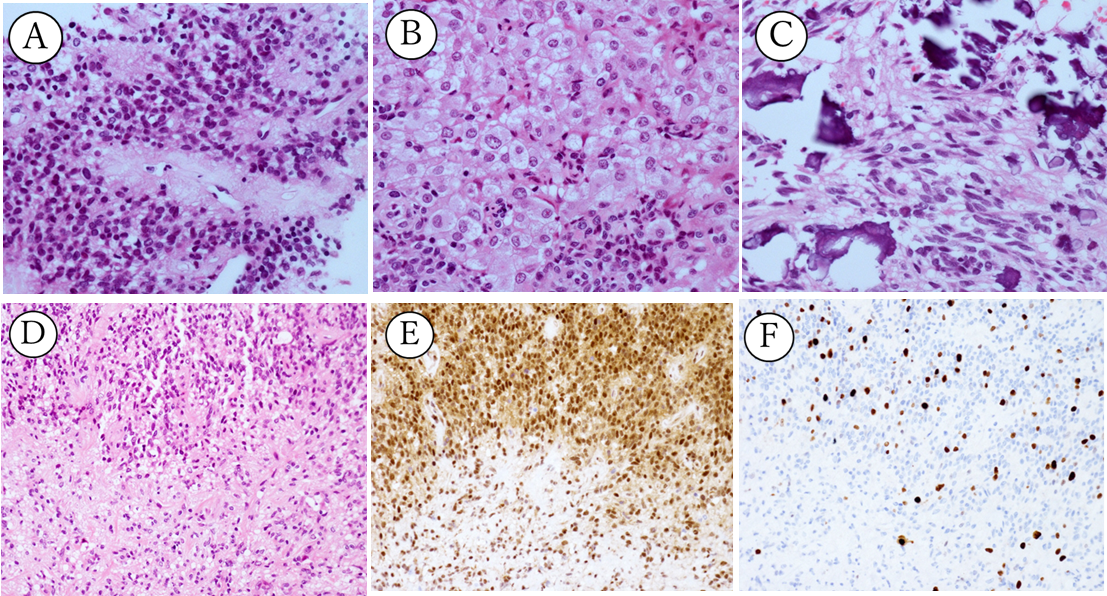


Supplemental Fig. 2

Supplemental Fig. 2


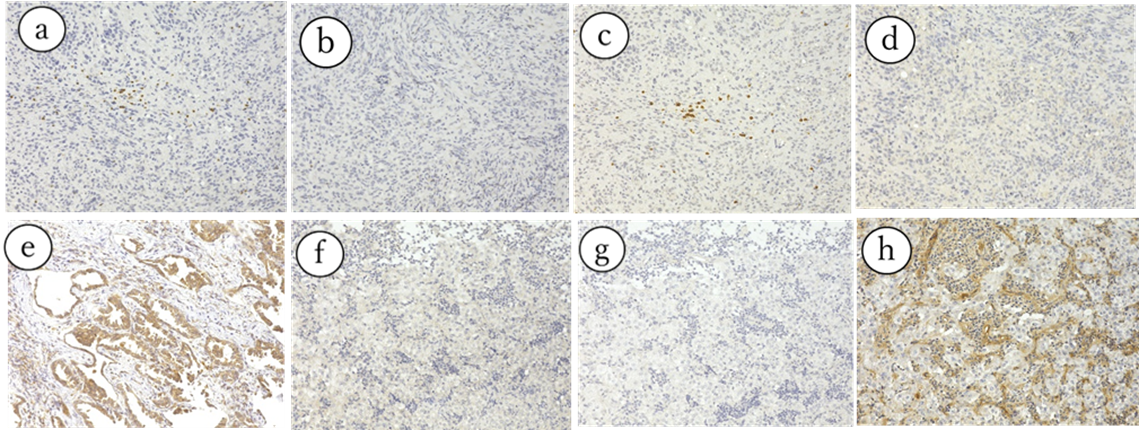

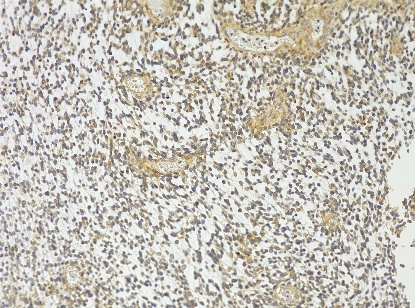

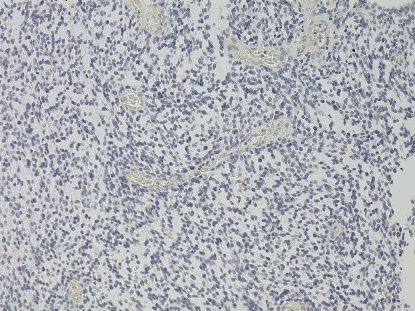

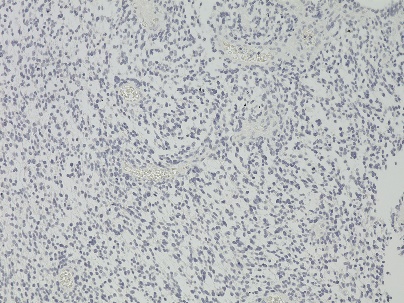

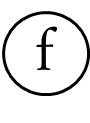

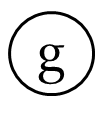

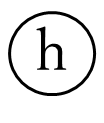


Supplemental Fig. 3

Supplemental Fig. 3


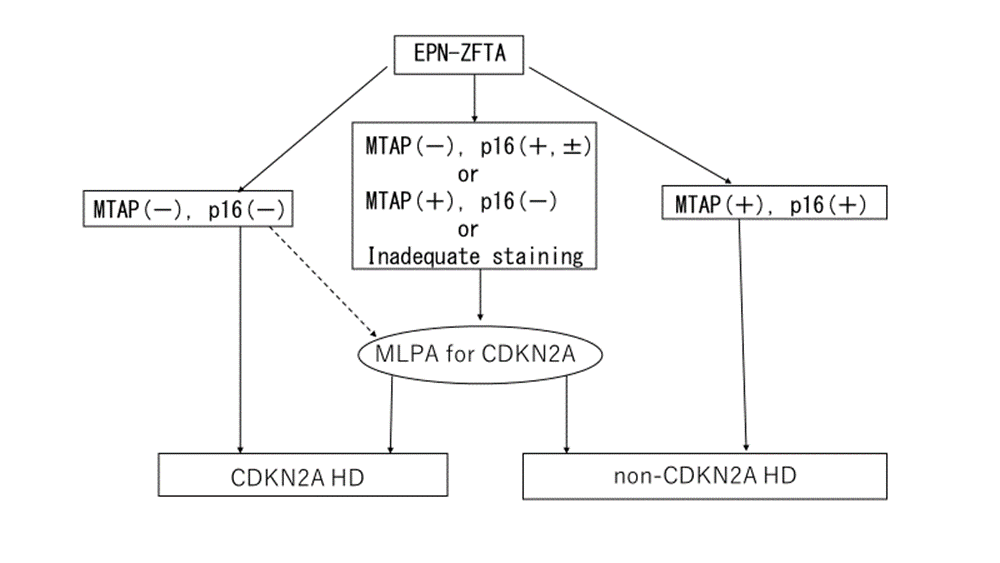


Supplemental Fig. 4
